# First record of experimentally induced salmon gill poxvirus disease (SGPVD) in Atlantic salmon (*Salmo salar* L.)

**DOI:** 10.1186/s13567-020-00787-9

**Published:** 2020-05-07

**Authors:** Even Thoen, Haitham Tartor, Marit Amundsen, Ole Bendik Dale, Karoline Sveinsson, Hans Petter Rønning, Estelle Grønneberg, Maria Krudtå Dahle, Mona Cecilie Gjessing

**Affiliations:** 1 Department of Fish Health and Welfare, Norwegian Veterinary Institute, PO Box 750 Sentrum, 0106 Oslo, Norway; 2 Sisomar AS, Trollbukta, 8226 Straumen, Norway; 3 Department of Molecular Biology, Norwegian Veterinary Institute, PO Box 750 Sentrum, 0106 Oslo, Norway

## Abstract

Salmon gill poxvirus (SGPV) infection is a common denominator in many cases of complex gill disease in the Norwegian salmon farming industry and may, as a single agent infection, result in salmon poxvirus disease (SGPVD). Experiences from the field suggest that stress may be a decisive factor for the induction of SGPVD. Here we investigated the effect of stress hormone treatment on SGPV kinetics and disease development. In our experiment, Atlantic salmon were divided into four groups. Two groups of fish received an intraperitoneal injection of hydrocortisone dissolved in a fatty vehicle, whereas fish in the other two groups received a sham injection of the vehicle. After 24 h, one group with hydrocortisone injection and one with sham injection were exposed to dead SGPV-infected fish. Plasma cortisol level, virus kinetics, virus localization, and pathological gill were monitored for 4 weeks post-exposure. Hydrocortisone injected fish displayed higher plasma cortisol and SGPV loads than non-hydrocortisone treated fish. Signs of SGPVD and ensuing mortality appeared only in fish exposed to the virus and injected with hydrocortisone around 2 weeks post-exposure. No clinical signs of disease or mortality were recorded in the other groups. Further, gill histopathology in diseased fish correlated well with SGPV load, with the infection apparently confined to gill epithelial cells. The current findings suggest elevated plasma cortisol being a prerequisite for the development of SGPVD and recommend minimization of stressful farming activities, particularly if SGPV infection has been previously identified.

## Introduction

Salmon gill poxvirus (SGPV) has been confirmed as a key pathogen responsible for gill disease in farmed Atlantic salmon [1–3]. While the specific pathologies and disease problems associated with SGPV infection have been observed for more than 20 years, the virus was first described by Nylund et al. in 2008 [4]. Following the characterization of the SGPV genome and subsequent development of molecular diagnostic tools, the impact of the virus in terms of prevalence and correlation with clinical disease and pathological change has become evident. Screening for SGPV has demonstrated that the infection is prevalent in Atlantic salmon and that covert infections are common. SGPV is found in several salmon producing countries, including Norway, Scotland, and the Faroe Islands [1]. In Canada, a virus similar to—but not identical with—the European SGPV genotypes has also been detected [5]. The Canadian sequence had a partial genome coverage of 29% and a genetic similarity of 79% (on average) when compared with the full Norwegian SGPV genome [5]. Natural infection with North European SGPV isolates is usually associated with apoptotic gill epithelial cells resulting in a reduced functional respiratory surface, and on occasion high, acute mortality [3]. However, in order to determine the causal relationship between SGPV infection and the associated disease, Koch’s second and third postulates should be fulfilled [6]. This implies that the virus should be first isolated from SGPV-infected fish, cultivated in vitro, then used to induce the typical disease signs in a previously healthy organism. Despite the reported infection of a Canadian isolate of SGPV in chinook salmon embryo (CHSE) cells [5], no successful cultivation studies of SGPV have been published. However, we know from different pilot studies that transmission of SGPV from infected to naïve fish, without clinical signs of disease, is possible both experimentally and in the field.

Several pathogenic agents isolated from diseased individuals do not reproduce clinical disease when used in experimental trials. This has been reported for experimental-infection studies with infectious pancreatic necrosis virus (IPNV) [7], *Piscine orthoreovirus* (PRV) [8], and *Saprolegnia* spp. [9]. However, in two of these studies [7, 9], when the fish were debilitated by intraperitoneal injection of cortisol, signs of clinical disease could be observed. Cortisol, produced by the interrenal tissues, is the major corticosteroid of teleost fish [10]. High plasma cortisol is commonly used as a chronic stress indicator in salmonids [11, 12] and has been linked to immunosuppression in fish as reviewed by Wendelaar Bonga [13]. Fish gills have been shown to be among the main target tissues for cortisol [14, 15]. Cortisol treatment was shown to affect energy, metabolism and salinity tolerance in the gills of gilthead seabream [14] and Atlantic salmon [15], respectively. In the current study, we hypothesize that a similar principle might also apply to induce clinical disease following SGPV infection in Atlantic salmon. Therefore, we designed an experiment in which hydrocortisone was injected to induce stress in fish prior to exposure to the virus. To the best of our knowledge, the current study presents the first experimental infection model for SGPV.

## Materials and methods

### Fish and experimental design

**Figure 1:**
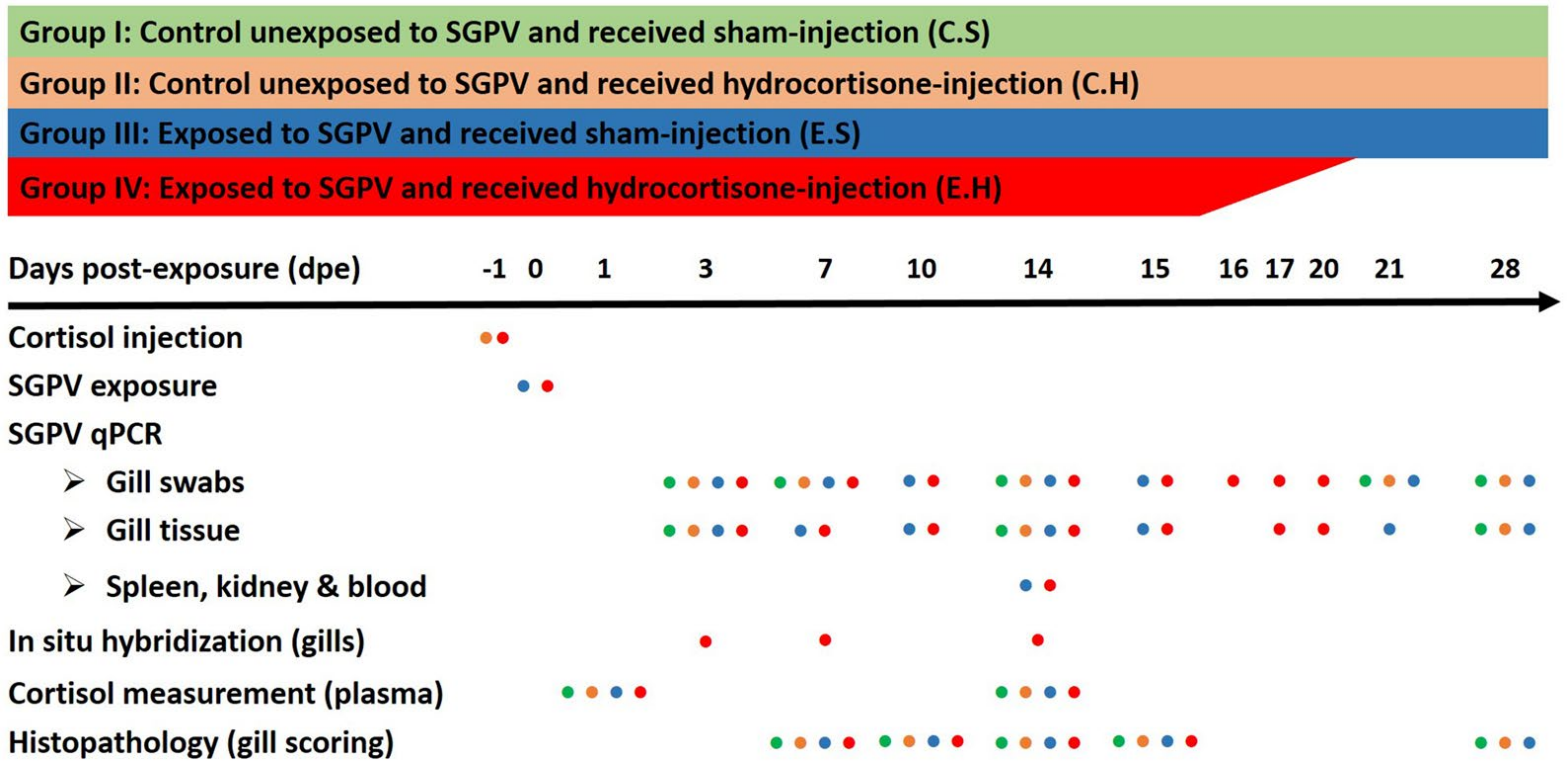
**Schematic representation of SGPV infection trial and analyses performed.** The colored dots represent groups that have been treated or analyzed in each experiment at particular time points.

Atlantic salmon (*Salmo salar*) pre-smolts (*n* = 220; average body weight 50 g) were used. Fish were acclimated in freshwater at 12 °C with the photoperiod maintained at a constant 14 h light/10 h dark cycle and were fed a 1% (w/w) daily ration of fish feed. After acclimation, the fish were randomly divided into four groups (I–IV), and the groups were allocated into 4 different 500 L tanks (55/each). To investigate the effect of increased blood cortisol hormone on salmon susceptibility to SGPV infection, fish in two groups (II and IV) were injected intraperitoneally with hydrocortisone. As a control for hydrocortisone injection, fish in the other two groups (I and III) received a sham injection at the same time. After 24 h, one group with sham injection (III) and one with hydrocortisone injection (IV) were exposed to dead SGPV-infected salmon, while groups I and II were left unexposed to the virus as a control. The exposure to the virus was accomplished by placement of ten SGPV-infected dead Atlantic salmon (average body weight = 150 g) with naïve fish in each experimental tank of groups III and IV for 24 h, with biomass ratio equals approximately to 1:1.83. The SGPV-infected dead fish placed in groups III and IV had an average SGPV Ct values in gill swabs equals to 21.2 and 21.4, respectively. The SGPV-infected fresh dead fish were shipped overnight on ice to the trial facility from an SGPVD outbreak in Northern Norway. The I, II, III and IV groups will, from here on, be referred to as C.S (control unexposed and received sham injection), C.H (control unexposed and received hydrocortisone injection), E.S (exposed and received sham injection), and E.H (exposed and received hydrocortisone injection), respectively. Because of the sudden appearance of the SGPV outbreak, it was not possible to have tank replicates for each of the four treatments in the current experiment. To reduce the impact of fish handling and anesthesia on the resting level of plasma cortisol, fish were sedated in their holding tanks using 2 mg/mL iso-eugenol (AQUI-S^®^ vet., Scan Aqua AS, Norway) before being anesthetized with 10 mg/mL of the same component. When euthanasia was necessary during the experiment, an overdose of iso-eugenol (30 mg/mL) was used to terminate the fish. An overview of the experimental design, samples collected, and analyses performed at different time points is shown in Figure 1.

### Preparation and administration of hydrocortisone depots

Hydrocortisone injections were prepared by mixing 10 mg hydrocortisone (Sigma H401) with 1 mL of a vehicle (a mixture of rapeseed oil and coconut shortening 1:1 [v:v]), as described by Gadan et al. [7]. A total volume of 100 µL of the cortisol-vehicle preparation was administered intraperitoneally into anesthetized fish assigned for cortisol injection (C.H and E.H groups), resulting in an approximate concentration of 20 µg hydrocortisone g^−1^ fish body weight. To standardize individual hydrocortisone doses as far as possible, the hydrocortisone-vehicle mixture was kept liquid and stirred during the injection process using a stirring hot plate (40 °C).

### Sampling

Five fish were anesthetized from each of the four experimental groups (C.S, C.H, E.S, and E.H) at 1, 3, 7, 10, 14, and 15 days post-exposure (dpe) and sampled for different analyses as shown in Figure 1. Moribund and dead fish in the E.H group were collected daily between 15 and 20 dpe. Moribund fish were euthanized, and all fish were sampled with the exception of dead fish displaying signs of autolysis. At later time points (21, 28 dpe), five fish were sampled from the C.S, C.H, and E.S groups only (there were no longer fish available in the E.H group due to the early mortalities). Gill samples were also taken from the fish population from which the virus shedders originated.

Gills from experimental fish and the field outbreak were swabbed with cotton swabs (Sarsted, GE) and the swabs were frozen (−80 °C) on RLT lysis buffer (Qiagen Inc., Valencia, CA, USA) until analysis for SGPV by qPCR. Blood samples were collected from the caudal vein of euthanized fish using vacutainer tubes (Vacutest, Sarstedt) coated with sodium heparin and 20 G Venoject needles. The tubes were centrifuged at 3000 × *g*, 4 °C for 10 min to separate plasma from blood cells. The plasma and blood cell pellets were frozen at −80 °C until further analysis.

Gills, spleen, and head kidney tissues were collected in formalin for histopathology and in situ hybridization, and in RNAlater (Qiagen Inc., Valencia, CA, USA) for SGPV qPCR analysis. To minimize the risk of virus contamination between organs during sampling, new equipment was used for each organ, and fish from groups unexposed to the virus (C.S and C.H) were always sampled first. Virus-contaminated equipment was washed in water, soaked in a Virkon solution (Virkon Tablets Rely^+^ON™) for 10 min, soaked in water again, and finally bathed in 70% ethanol for 5 min.

### DNA extraction and qPCR for SGPV

DNA was extracted from gill swabs, blood pellets, gill, spleen and kidney tissues and qPCR was performed to detect SGPV DNA. DNA was extracted from gill swabs, using the MagNA pure 96 system and Viral DNA Large Volume kit (Cat. No. 6374891001, Roche Applied Science), following the manufacturer’s Pathogen Universal 500 3.1 protocol. The gill swab was put into 500 µL of RLT buffer and the extracted DNA was eluted in 50 µL of elution buffer. DNA extraction from gills, spleen, kidney, and blood was performed as described by Gjessing et al. [3], using a QIAcube^®^ and QIAamp DNA mini kit (51326, QIAGEN, Hilden, Germany). A total amount of 20 mg of each tissue sample was lysed at 56 °C in a mixture of 180 µL ATL buffer and 20 µL Proteinase K. Following DNA isolation, 1 µL (1 mg/mL) of ribonuclease A (Sigma Aldrich) was added per 100 µL extracted DNA to remove traces of RNA. The quantity and purity of the extracted DNA were measured using a NanoDrop™ 2000 spectrophotometer (Thermo Scientific, Wilmington, DE, USA). A qPCR assay directed against the SGPV D13L genomic sequence was run using a Stratagene system (Agilent) and related software (MxPro-Mx3005P), as described previously [3]. Virus-specific primers and probe were employed with the following PCR parameters: 50 °C for 2 min (UDG incubation), 95 °C for 15 min (UDG inactivation) and 45 cycles of 94 °C/15 s, 55 °C/30 s and 72 °C/15 s were used. Cycle threshold (Ct) values ≥ 40 were considered negative. Groups and time points analyzed with qPCR are shown in Figure 1.

### Histology and in situ hybridization

Formalin-fixed paraffin-embedded (FFPE) sections from gills, spleen, and kidneys were prepared and stained with hematoxylin and eosin (H&E) according to standard protocols and examined for any histopathological alterations. The degree of apoptosis in gill epithelia was evaluated semi-quantitatively. The evaluation resulted in a score ranging from 0 to 3, where 0, 1, 2 and 3 indicated no, mild, moderate and extensive degree of apoptosis, respectively.

Serial sections to those analyzed for histopathological alterations were used for in situ hybridization analysis (ISH) to confirm the virus load indicated by qPCR and to study cell tropism and distribution of SGPV in gill tissues. An ISH protocol based on RNAscope technology was applied using RNAscope^®^ 2.0 HD Red Chromogenic Reagent Kit (Advanced Cell Diagnostics Inc.). In this method, paired double-Z oligonucleotide probes (V-SGPV-098, Cat. No. 540201), targeting nucleotides (97744–98794) on the mRNA of the SGPV D13L gene (SGPV098; accession number: GenBank KT159937) were used. A negative control probe (dapB, Cat. No. 310043) targeting the bacterial dapB gene was employed to assess background signals. In addition, a positive control probe (ppib, Cat. No. 494421) targeting salmon peptidylprolyl isomerase B gene (Accession number: GenBank 213515059) was also used. All probes were designed using custom software, as described by Wang et al. [16]. RNAscope was performed following the manufacturer’s instructions, on gill tissue samples collected at 3, 7, and 14 dpe. Briefly, FFPE sections were deparaffinized in xylene and rehydrated through a series of alcohol washes. The rehydrated sections were then treated with hydrogen peroxide at room temperature for 10 min to block endogenous peroxidases. The sections were then boiled in target retrieval buffer for 15 min and incubated with protease at 40 °C for 15 min. The slides were then hybridized with the probes specified earlier at 40 °C for 2 h and then run through a sequence of signal amplification (40 °C for 15 or 30 min) and washing steps. Finally, the hybridization signal was visualised using Fast Red. All slides were counterstained using Mayer’s hematoxylin (Chemi Teknikk, 5B-535) diluted in distilled water 1:1 (vol/vol) for 2 min.

### Plasma cortisol measurements

The cortisol concentration was measured in plasma samples of fish (*n* = 5/group) from groups with (C.H and E.H) or without (C.S and E.S) injected hydrocortisone depots, using a monoclonal antibody enzyme-linked immunosorbent assay (ELISA) (Enzo Life Sciences, Inc., Farmingdale, NY, USA). In brief, 100 μL of plasma diluted 100× in assay buffer, was applied to 96-well microtiter plates coated with anti-Mouse IgG. To determine intra-assay precision, a series of twofold diluted standard cortisol samples (100 μL) from 10 000 to 156 pg/mL was run on each plate. A total volume of 50 μL assay buffer, 50 μL cortisol ELISA enzyme-conjugate, and 50 μL cortisol antibody were added successively to appropriate wells according to the manufacturer’s instructions. The cortisol in the sample would bind competitively with the enzyme conjugated to cortisol. The plates were then incubated at room temperature on a plate shaker for 2 h at 500 rpm. After incubation, the plates were washed 3 times using 400 μL washing buffer. After the final wash, 5 μL of a blue conjugate (included in the kit) was added to the appropriate wells. To detect the enzyme activity in all wells, 200 μL pNpp substrate solution was added, and the plate was incubated at room temperature for 1 h without shaking. Stop solution (50 μL) was used to stop the enzymatic reaction, and the optical density was read at 405 nm using an ELISA plate reader (Multiscan EX, Artisan). The enzymatic activity results in an inverse relationship between optical density and the amount of cortisol in the sample. This inverse relationship was calculated using a standard curve established from cortisol standard samples.

### Statistics

A Wilcoxon signed-rank test was used to determine the significance of the differences in mean level of plasma cortisol between C.H and C.S, and between E.H and E.S. JMP software (JMP^®^, Version 11, SAS Institute Inc., Cary, NC, 1989–2007) was used for the statistical analysis with the α value set to 0.05. GraphPad Prism (GraphPad Software) was used to make the graphs presented in the current work.

## Results

### Confirmation of clinical SGPVD and SGPV infection

**Figure 2:**
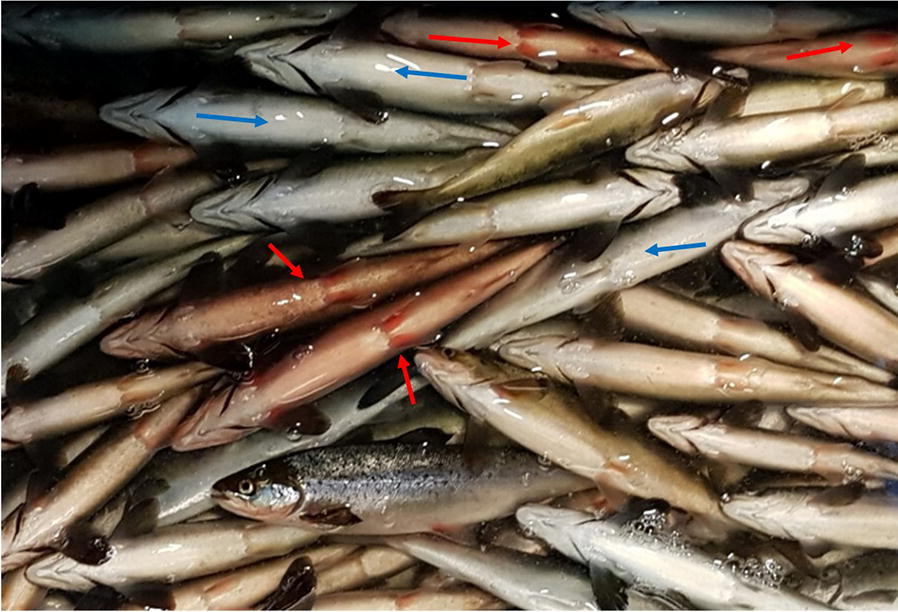
**Observations on Atlantic salmon collected during SGPVD outbreak.** Photo of dead and moribund salmon showing fish with skin and fin redness (red arrows) or with normal skin color (blue arrows). Regardless of skin color, all fish were confirmed to be SGPV positive (Median Ct values of 21.04) and were used as infectious material in the current study.

Atlantic salmon fish obtained from an acute disease outbreak in a juvenile Atlantic salmon production facility in Nordland, Norway in 2019, showed signs of lethargy, loss of appetite, and respiratory distress. These fish were collected from one tank, as the cumulative mortality had reached 45%. Postmortem examination revealed redness in the skin of some fish, most noticeable in the non-pigmented areas of the ventral surface and at the basis of the pectoral and abdominal fins (Figure 2). Raised hemoglobin levels were found in the plasma of fish with red skin. qPCR analysis revealed SGPV Ct values ranging between 17.94 and 23.38, with a median of 21.04. The fish displayed histopathological changes in the gills typical for SGPVD, including extensive apoptosis of gill epithelial cells, and were thereby confirmed to suffer from SGPVD. Fish from this outbreak were used as a source of infection in the experimental trial.

### Clinical SGPVD and mortalities were reproduced experimentally only in fish exposed to SGPV and injected with hydrocortisone

**Figure 3:**
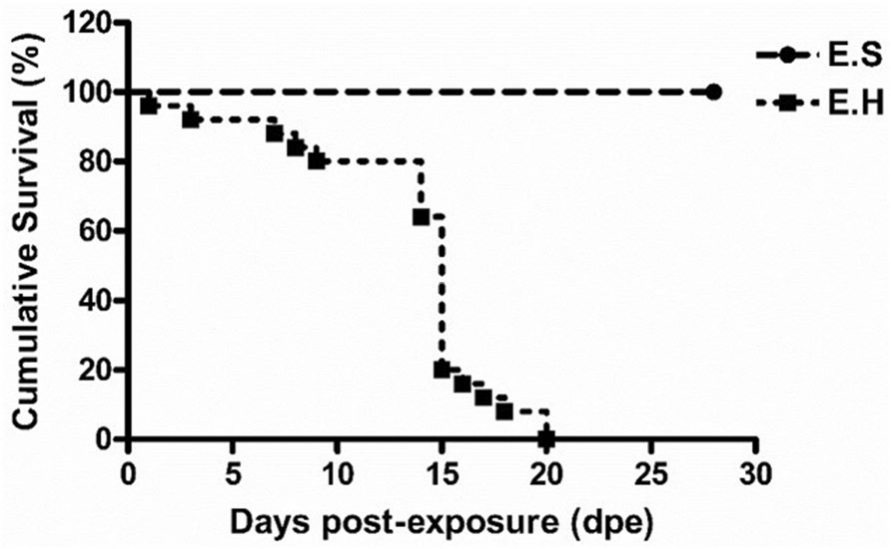
**Mortality due to SGPV infection in fish with and without hydrocortisone depots.** Cumulative Kaplan–Meier survival curve of Atlantic salmon fish in groups exposed to SGPV (E.S; exposed to SGPV and received sham injection and E.H; exposed to SGPV and received hydrocortisone injection) in the SGPV experimental infection trial following exposure to dead fish obtained from an SGPVD outbreak.

The experimental fish were monitored daily for SGPVD signs and mortality in the same tank they were sampled from due to the lack of tank replication. Single mortalities (dead or humanely euthanized) were registered at 1, 3, 7, 8, and 9 days post-exposure (dpe) in the E.H group (Figure 3). On 14 dpe, fish in the E.H group began to display lethargic swimming, loss of appetite, and respiratory distress, and four new mortalities were registered. One day later, the signs of respiratory distress in the E.H group increased, sluggish behavior was noticed, and mortality peaked (11 fish died or were humanely euthanized; Figure 3). In addition, a few fish in the E.H group, although still alive, appeared rigid (i.e., “live rigor”). The remaining fish in this group were either humanely euthanized or died during the following week. Monitoring fish mortalities in tanks assigned for sampling means that the numbers of fish used to calculate survival rates at 3, 7, 10, 14, 21 and 28 dpe were 5 fish less than that used to calculate survival rate at 1, 3, 7, 10, 14, and 21 dpe, respectively. No clinical signs of SGPVD or mortalities were observed in the other groups (E.S, C.H and C.S) at any time.

### SGPV gill tissue load corresponded with the clinical course of disease and gill epithelial cell apoptosis score

**Figure 4:**
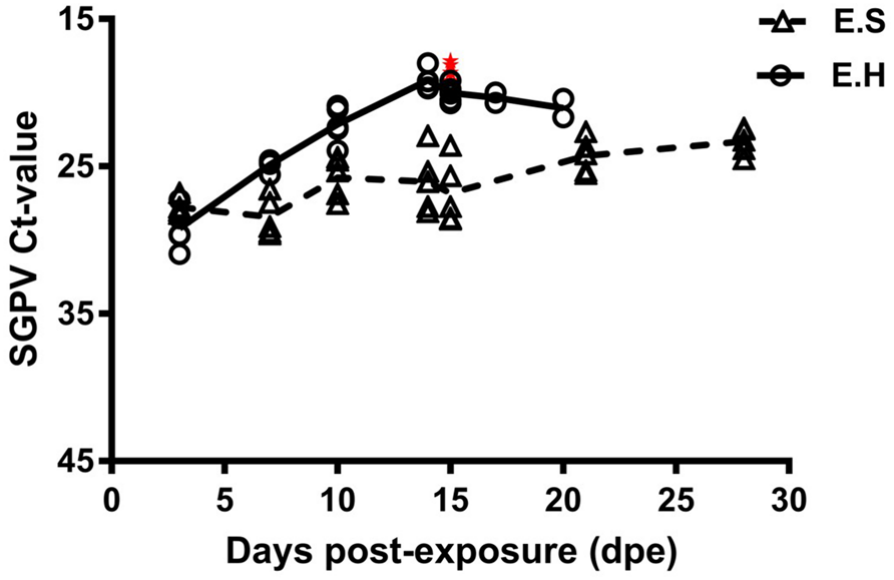
**Intraperitoneal deposition of hydrocortisone increases the susceptibility of Atlantic salmon to SGPV.** SGPV Ct values (assay targeting the SGPV D13L gene, Median with range) obtained from gills from fish in different groups (E.S; exposed to SGPV and received sham injection, and E.H; exposed to SGPV and received hydrocortisone injection) at different time points following virus exposure. Moribund fish in the E.H group are shown as red stars in the Figure.

SGPV infection of fish in the E.S and E.H groups was confirmed by SGPV specific qPCR. Fish in the E.S. group displayed SGPV Ct values ranging from 22.42 to 29.61 with a median of 26.07 while the values of fish in the E.H group ranged between 17.86 and 30.93 with a median of 20.59, throughout the 4-week experimental period. Fish in the C.S and C.H groups tested negative for the presence of SGPV throughout the trial (Figure 4).

**Figure 5:**
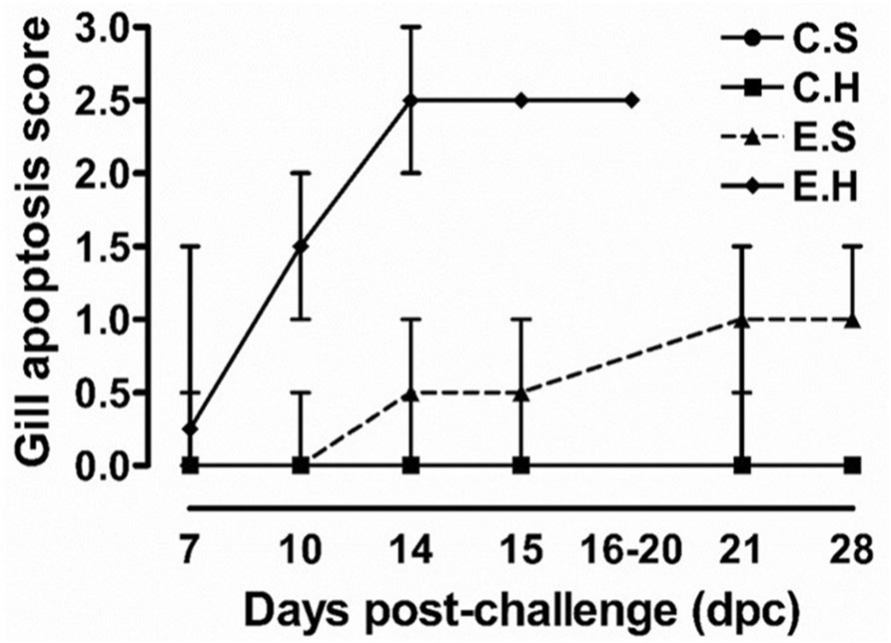
**Hydrocortisone depot exaggerates SGPV-mediated pathology.** Gill epithelial cell apoptosis score (median with range) of fish (*n* ≥ 5) in different groups (C.S, C.H, E.S, and E.H) during the SGPV experimental infection course.

As early as 3 dpe, SGPV median Ct values of 28.03 and 29.63 were identified in the E.S and E.H groups, respectively (Figure 4). Gill epithelial cell apoptosis was not observed at this time (Figure 5). An increase in SGPV load was identified at 7 dpe in the E.H group (median Ct value of 24.73) but not in the E.S group (median Ct value 29.11). Fish in both E.S and E.H groups (*n* = 5 in each), displayed a slight degree of gill epithelium apoptosis with a median apoptosis score of 0 and 0.25, respectively (Figure 5).

At later time points, the difference between the two groups increased continuously. Fish analyzed in the E.S and E.H groups had lower median SGPV Ct values at 10 dpe (25.34 and 22.34), 14 dpe (26.07 and 19.44), and 15 dpe (27.75 and 19.52) than those obtained earlier in the trial (Figure 4). Accordingly, all the gills collected from fish in the E.H group at 10 (*n* = 5), 14 (*n* = 6) and 15 (*n* = 12) dpe showed high median scores for gill epithelial cell apoptosis (1.5, 2.5, and 2.5; respectively). Gill epithelial cell apoptosis analysis (represented by ratio of fish with gill epithelial cell apoptosis/total fish; median apoptosis score) of fish in the E.S group was (2/5; 0), (3/5; 0.5), and (3/5; 0.5), at 10, 14 and 15 dpe, respectively. These results demonstrate a clear difference between the two groups, which indicates an increased susceptibility to infection and disease in hydrocortisone injected fish and once again confirms previous observations that qPCR enumeration (Ct values) of viral DNA correlates with gill epithelial cell apoptosis score.

Between 16 and 20 dpe, a median SGPV Ct value of 20.55 was identified in dead and moribund fish in the E.H group (Figure 4), with all the fish examined (*n* = 6) displaying a significant degree of gill epithelial cell apoptosis (median score = 2.5). Higher loads of SGPV were also identified (median Ct values of 24.19 and 23.26 at 21 and 28 dpe respectively, Figure 4) in gill tissues sampled from fish in the E.S group, as were increased levels of gill epithelial cell apoptosis (median score = 1 for both groups) compared to earlier time points. Again, this result is in accordance with the trend of the more SGPV DNA detected, the higher the score of gill epithelial cell apoptosis observed.

### Increased plasma cortisol was confirmed in hydrocortisone-injected fish

**Figure 6:**
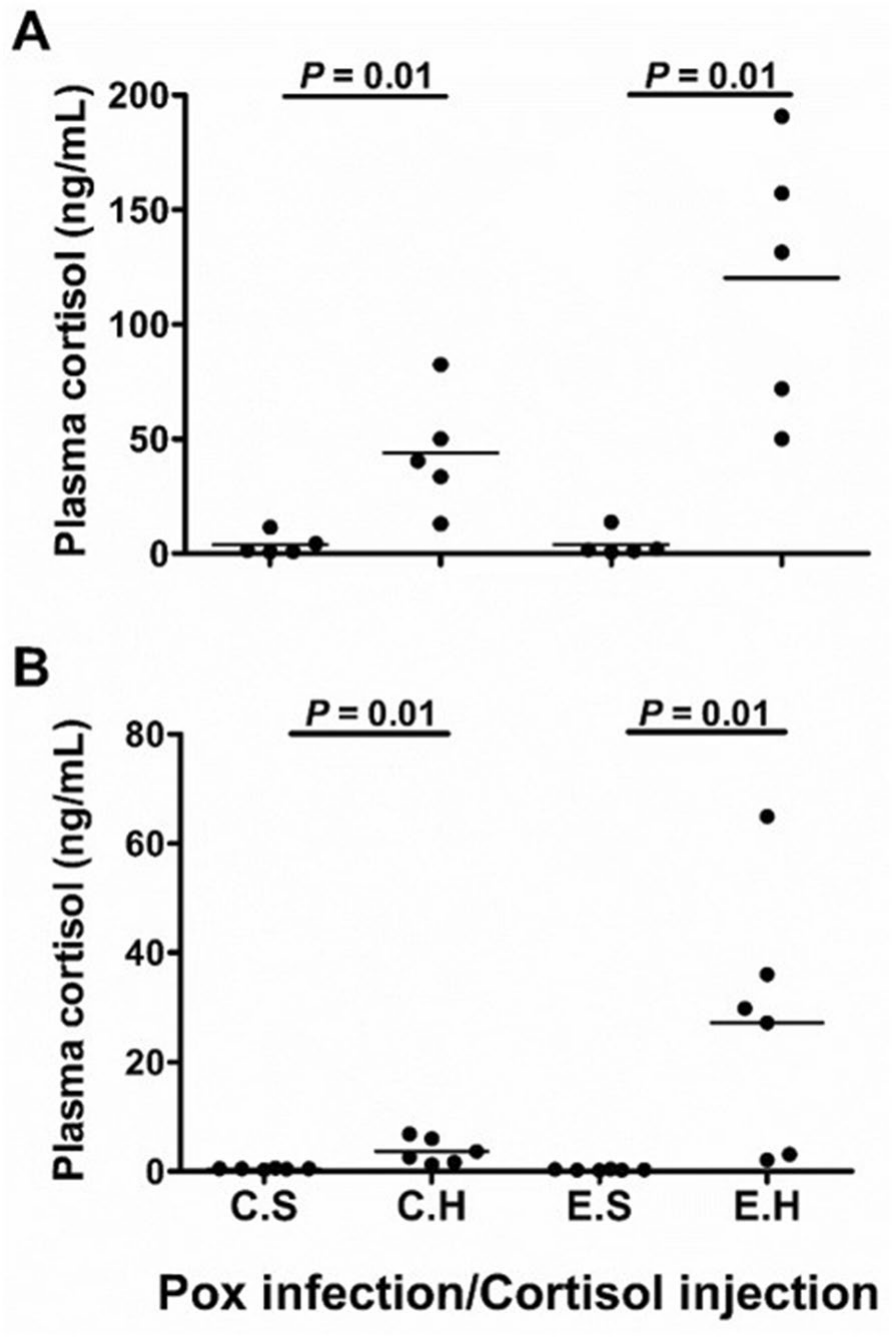
**Intraperitoneal injection of hydrocortisone elevates plasma cortisol levels in Atlantic salmon.** Dot blots of plasma cortisol concentration (ng/mL) in fish collected from different experimental groups (C.S, C.H, E.S, and E.H) in SGPV experimental infection trial at **A** 1-day post-exposure (dpe) and **B** 14 dpe. Each plasma sample is represented by a dot, and horizontal lines in the blot indicate the median per group. The Wilcoxon test was used to determine statistical significance between the groups with bars representing *P*-values. Differences between groups were considered statistically significant when *P *< 0.05.

To link stress hormone treatment in fish to the presence or absence of clinical signs and ensuing mortalities after virus exposure, we measured the concentration of plasma cortisol (ng/mL) in all groups (*n* = 5 per group). Our results showed that the average plasma cortisol level in hormone-treated fish (C.H and E.H groups) were significantly higher than in the groups without hormone treatment at 1 and 14 dpe (*P* = 0.01 for all; Figure 6). The average plasma cortisol concentration in the E.H group at 1 and 14 dpe (120.23 and 27.19 ng/mL) was higher than for the C.S (3.7 and 0.4 ng/mL), C.H (43.8 and 3.6 ng/mL) and E.S (3.8 and 0.3 ng/mL) groups, respectively (Figure 6).

### SGPV infection is confined to the gill epithelium

**Figure 7:**
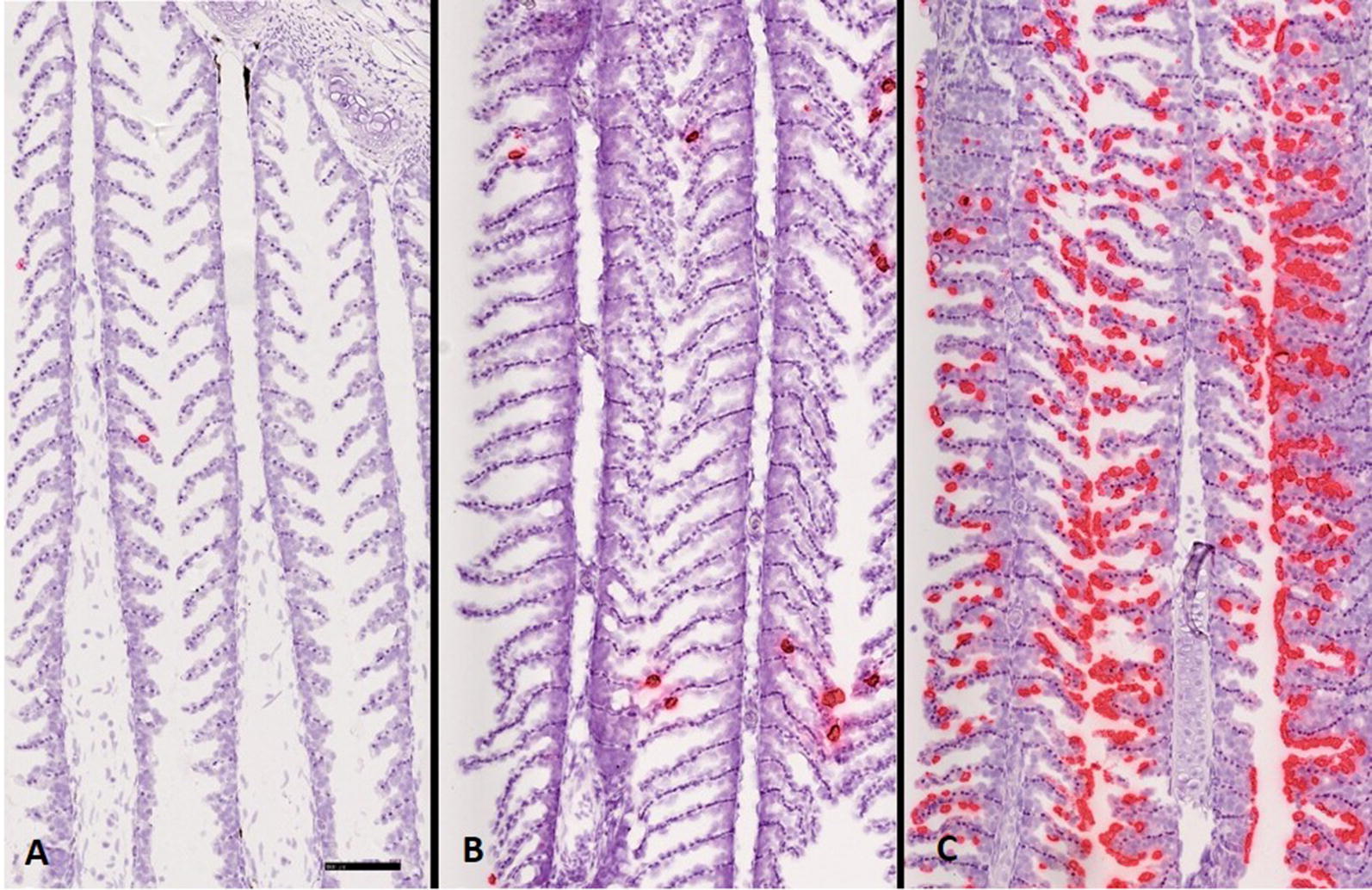
**In situ** **hybridization (mRNA) of gill sections collected from Atlantic salmon exposed to SGPV.** Fish (from E.H group) injected intraperitoneally with hydrocortisone and exposed to SGPV 1 day after injection. In situ hybridization performed on gills sampled at **A** 3, **B** 7, and **C** 14 days after virus exposure showed a gradual increase in virus load and infected gill epithelium cells (in red) over the course of infection. Scale bar = 100 μm.

To demonstrate the distribution and cell tropism of SGPV in gill tissues, in situ hybridization utilizing RNAscope was performed on gill tissues from the E.H group at 3, 7, and 14 dpe. At 3 dpe, a few gill epithelial cells showed positive staining for SGPV (Figure 7A). In line with rapid virus replication, more gill epithelial cells became apoptotic and were positive for SGPV at 7 dpe (Figure 7B). At 14 dpe, the number of gill epithelial cells staining positive for SGPV had increased and dominated most of the gill section (Figure 7C). SGPV-specific staining was confined to the gill epithelium at all times. No staining was identified elsewhere in the gills. The number of cells staining positively at any time-point correlated well with SGPV Ct value and gill epithelial cell  apoptosis score.

### Traces of SGPV were found only in some spleen, kidney, and blood samples collected from experimentally-infected fish

Although SGPV infection appears to occur primarily in gill tissues, we wanted to investigate the spread of SGPV to other organs. Blood samples, kidney, and spleen tissues taken from fish (*n* = 5/group) in the E.H and E.S groups during the infection peak in gills (14 dpe) were analyzed for SGPV DNA using qPCR. Blood and spleen samples tested either negative or showed high Ct values. In the E.S group, kidney tissues from three of five fish were positive for SGPV with Ct values ranging between 32.75 and 38.07 (median Ct value 34.88). In the E.H group, four of five kidney samples were positive for SGPV with Ct values ranging between 31.87 and 37.40 (median Ct value 35.83). SGPV was also identified in spleen tissues of two of these four fish at Ct values of 34.24 and 36.44. The blood cells collected from fish in the E.S and E.H groups at 14 dpe tested negative for the presence of SGPV.

### Gill swab analysis reflects SGPVD infection state

We compared SGPV load (Ct values) in gill swabs and gill tissue samples collected at different time points post virus exposure. Our results showed that both sample types revealed a similar virus load pattern throughout infection. Ct values from samples collected by gill swabbing were, however, generally lower than those from gill tissue samples. For the E.S group, SGPV Ct values from gill swabs ranged between 21.74 and 29.63 from 3 to 28 dpe, with a median of 25.2 compared to 22.9 and 29.6 with a median of 27.5 obtained from gill tissues during the same period. Ct values from the E.H group were, in general, lower than that in the E.S group. Gill swab SGPV Ct values ranged from 15.36 to 29.36 with a median of 18.1, while Ct values in gill tissue samples ranged between 17.86 and 30.92 with a median of 20.5.

## Discussion

Salmon gill poxvirus disease (SGPVD) is becoming a serious problem in the Norwegian salmon farming industry. Studies related to the pathogenesis of SGPVD and the description of virus-host interactions are scarce due to the lack of an appropriate infection model. This study presents the first successful in vivo SGPV infection model in salmon recreating typical SGPVD signs and mortalities. Based on experience from field outbreaks and in common with a range of other infectious diseases [7, 9, 17, 18], stress seems to be an eliciting factor for the outbreak of SGPVD. We hypothesized, therefore, that clinical disease may be induced experimentally by treating fish with hydrocortisone prior to virus exposure.

The results of different pilot studies suggest that the treatment of SGPV infectious material obtained from clinical SGPVD is decisive for the outcome of subsequent infection trials. For example, infection of naïve fish using excised, intact or homogenized SGPV-infected gills or intact gills subjected to several freeze/thaw cycles, did not lead to established infections. Therefore, fish used as sources of infection in the current study were obtained from SGPV outbreak and were not processed but kept on ice overnight during shipping until the trial started. Introducing fish from the outbreak with similar infection levels to the experimental fish in the two E.H and E.S groups could guarantee similar exposure of fish in both groups to the virus. This could be inferred from the comparable SGPV Ct values detected in fish gill tissues collected from the E.H and E.S groups (Figure 4) collected at 3 dpe. Knowing that fish with high plasma cortisol level could release cortisol to the surrounding water environment [19], we kept the E.S and E.H groups in separate tanks to avoid any stress effect on fish in the E.S group that might be caused by cortisol released from the E.H.

In the present study, SGPVD has been reproduced experimentally by combining exposure to SPGV with stress hormone injection. Following exposure, SGPV replicated in the gills of fish in both groups exposed to the virus (E.H and E.S) and reached the peak of infection at 14 dpe. However, fish injected with hydrocortisone (E.H) allowed greater virus replication than fish without hydrocortisone treatment (E.S), as judged by mortalities, SGPV Ct values, and gill epithelial cell apoptosis score. Mortality was observed only in fish in the E.H group with the diseased fish showing signs of respiratory stress, loss of appetite, lethargic swimming, as well as severe gill epithelial cell apoptosis with low SGPV Ct values prior to death. These clinical signs, typical of SGPVD [1], demonstrate that we have successfully reproduced SGPVD experimentally.

In the current study, fish injected with hydrocortisone (C.H and E.H) showed higher plasma cortisol levels than fish injected with the vehicle only (C.S and E.S), respectively, at 1 and 14 days after virus exposure. The average of plasma cortisol levels in fish in the C.H (43.8 ng/mL) and E.H (120.23 ng/mL) groups at 1 dpe were in the range of plasma cortisol level measured in salmon during natural stress conditions [20, 21]. However, the difference in fish plasma cortisol between the E.S and E.H before virus exposure could be inferred from the difference between the C.S and C.H groups exposed to no virus, suggesting that fish in the E.H group had high plasma cortisol and were stressed at the time of virus exposure. As fish in all groups were pre-sedated with iso-eugenol [22] prior to injection, we believe that the high plasma cortisol level identified in E.H and C.H groups could not be caused by stress effect of fish handling. However, the significantly higher plasma cortisol level in fish in the E.H group compared to that in the C.H group could be attributed to extra stress caused by the virus infection in the E.H group. We suggest that the high load of exogenous cortisol in stressed fish in the E.H group might have paved the way for SGPV to induce more endogenous cortisol in this group. This suggestion is in accordance with the results of a previous study on stress and IPNV infection in salmonid fish, in which IPNV infection was found to trigger increased plasma cortisol level in fish with cortisol depots [7].

The higher susceptibility of fish with hydrocortisone depots (E.H) to SGPV in comparison to those without hydrocortisone (E.S) is consistent with our hypothesis that stress induced by cortisol increases the chances of SGPVD development in fish exposed to SGPV. During stress, fish defense mechanisms may be compromised and probably less able to combat infections [13]. Such an argument is consistent with previous findings showing that high levels of plasma cortisol in stressed fish could result in immunosuppression related to both nonspecific and specific immune activities [23–25]. Antiviral immune response against IPNV, including the induction of the antiviral IFNα-1 pathway and Mx mRNA expression, was shown to be delayed in salmon fish injected with slow release cortisol implants [7]. To this end, we believe that a potential delay of antiviral response to SGPV in fish in the E.H group could be the reason for the SGPVD signs and mortalities identified in that group.

Gill swabs can replace gill tissue sampling for the detection of SGPV. As SGPV is detected mainly in the gills of salmon with SGPVD, it is expected that gills are the main organ shedding SGPV during an infection. In addition, gill swabs can also detect virus from external exposure prior to infection and replication. In the current study, we found that SGPV RNA could be visualized by ISH in histologically normal gill epithelial cells during the early stages of infection. However, following rapid replication of the virus, signs of epithelial cell apoptosis and extensive shedding of gill epithelial cells could be seen during the peak of infection [2]. In previous studies, such shed cells were confirmed to contain large numbers of virus particles [1, 3].

Shed cells packed with the virus may attach to gill mucus. In the current study, analysis of swabbed gill mucus collected from infected fish revealed SGPV Ct values even lower than those obtained from gill tissue samples. This difference in virus number between the gill swab and gill tissue samples was seen throughout the study. We believe, therefore, that samples collected by the gill swabbing method can be as efficient as gill tissue for SGPV detection. In addition, gill swabbing allows for non-lethal sampling [26]. Non-lethal gill swabbing could allow sequential sampling from the same fish for follow-up purposes in different experimental contexts.

Respiratory distress and lethargic swimming are typical signs of SGPVD [2]. Both behaviors were observed in fish from the field outbreak used as infection material, and in fish from the group exposed to SGPV and injected with hydrocortisone (E.H. group). Respiratory distress in infected fish may have led to hypercapnia and respiratory acidosis [27]. The respiratory acidosis associated with hypoxic and hypercapnic conditions is known to disturb the metabolism in fish muscles [28, 29], and the consequences of such physiological processes might have contributed to the observed muscular “live rigor” reported from some field outbreaks. Many dead and moribund fish display redness of the skin during SGPVD outbreaks. This feature was observed in a few fish in our study and may have been caused by hyperemia, congestion, or hemorrhage. Potential explanations for such circulatory disturbances occurring during infectious diseases could be that pathogen propagation interferes with the blood homeostasis [30]. High hemoglobin levels observed in the plasma of some fish with SGPV infection in the present study, suggest a certain degree of red blood cell hemolysis.

The absence of clinical signs and mortalities in fish without hydrocortisone injection in the current study and other pilot studies, despite the high virus load in their gills, may reflect a role for stress in the virus’s ability to induce typical SGPVD. In that regard, various farming activities with stress consequences can increase the likelihood of the onset of clinical SGPVD in fish farms [31]. Anecdotal evidence from field outbreaks suggests that stressful events often occur prior to SGPVD outbreaks. In that regard, stressful routines such as sorting, vaccination and/or vibration due to building activities must be kept to a minimum to reduce the impact of SGPV infection.

In conclusion, this study is the first to reproduce SGPVD experimentally using dead SGPV-infected fish as a source of infection and hydrocortisone treatment as a predisposing factor. Clinical signs of SGPV infection with ensuing mortality were prominent at 14 dpe only in fish treated with hydrocortisone prior to exposure to the virus. The increasing SGPV load in fish gills and gill swabs corresponded well with the disease course and gill epithelial cell apoptosis score. The association between cortisol and SGPVD morbidity and mortality suggests the importance of implementing cortisol analyses in SGPV infected fish. These findings imply that control of environmental stressors and reduced handling of infected fish is crucial for avoidance of severe SGPVD.

## Data Availability

The datasets used and/or analysed during the current study are available from the corresponding author on reasonable request.

## Abbreviations

C.S: control unexposed and received sham injection
C.H: control unexposed and received hydrocortisone injection
E.S: exposed and received sham injection
E.H: exposed and received hydrocortisone injection
IPNV: infectious pancreas necrosis virus
PRV: *Piscine orthoreovirus*
SGPV: salmon gill poxvirus
SGPVD: salmon gill poxvirus disease
